# Case Report in the Brazilian Context: Cognitive and Behavioral Changes Following an Electric Injury

**DOI:** 10.3389/fpsyt.2021.684817

**Published:** 2021-07-20

**Authors:** Katie Moraes de Almondes, Julianna Pinto de Azevedo, Marina Bruxel dos Santos, Walter Barbalho Soares

**Affiliations:** ^1^Neuropsychology of Aging Service, Onofre Lopes University Hospital, Department of Psychology, Federal University of Rio Grande Do Norte, Natal, Brazil; ^2^Neuropsychology of Aging Service, Department of Psychology, Federal University of Rio Grande Do Norte, Natal, Brazil; ^3^Psychosocial Care Unit, Onofre Lopes University Hospital, Federal University of Rio Grande Do Norte, Natal, Brazil

**Keywords:** electrical injury, dementia, neuropsychology, psychiatric symptoms, cognitive deficit, funcionality

## Abstract

Electrical injury (EI) is the sequel of an electrical shock. Physical sequelae are most common, but also other symptoms can happen, such as neurological symptoms, psychiatric alteration, and cognitive decline. The repercussion of EI can happen whether or not the head is a point of contact with the electrical current. There are no official diagnostic criteria for cognitive repercussions of EI, which may lead to incorrect diagnostics and confusion with other most frequent causes of dementia, such as frontotemporal dementia, pseudodementia, or dementias for reversible causes. In this case report, we described a right-handed man, aged 56 years old, referred to our service due to behavioral changes and cognitive alterations related to electric shock. The psychiatric team has monitored him, but cognitive deficits have raised doubts about the presence of dementia syndrome. The neuropsychological evaluation revealed severe deficits and loss of functionality, which filled the criteria for major neurocognitive disorder according to the Diagnostic and Statistical Manual of Mental Disorders fifth edition (DSM-5). Adding these findings to the patient's history and after a detailed investigation of other causes of dementia, we concluded that this is a possible case of EI with strong neuropsychological symptoms. This case report should help clinicians to recognize this condition and its features. We aimed to share the importance of recognizing the neuropsychological and psychiatric features of EI, mainly in the Brazilian context.

## Introduction

Electrical injury (EI) is the sequelae of industrial or residential accidents that involve electrical shock. Physical sequelae are common, such as burns, cardiac manifestations, or injuries due to falls (1–3). Neurological, neuropsychological, and psychiatric symptoms are also consistently found in cases of EI and may be present whether the head is a point of contact with the electrical current or not (4). Electrical injury is more prevalent in men, probably due to exposure to industrial environments and jobs at construction (3, 5). Physical and neurological sequelae have been studied more consistently in recent years, yet long-term cognitive and psychiatric disorders have not been described in the context of the Brazilian population.

One of the possible reasons for the high rates of neurological sequelae associated with electrical injuries could be the lower levels of resistance in blood vessels and nerves concerning bones and fat, which would facilitate the conduction of electrical current by the central nervous system (3, 6). However, many other factors can be related and influence the severity of the consequences, such as the touch voltage, type of current [direct current (DC) or alternating current (AC)], the duration and path of the current through the body, and others. Unfortunately, pathway effects on sequelae have not been extensively studied (3).

Electrical injury survivors have a high rate of psychiatric changes, most frequently presenting symptoms of depression, anxiety, posttraumatic stress disorder, difficulty in adjustment, and somatic preoccupation (7, 8) that persist and can predict the emotional and functional outcome in the postacute phase (8). Deficits in episodic and working memory, attention, executive functions, and visuospatial and motor abilities (4, 8–10) were previously described in the literature and are known to be possible in EI cases, despite no finding of a characteristic pattern of structural brain damage (9). A previous study (10) found that cognitive symptoms are independent of psychiatric disorders in EI survivors, supporting the hypothesis that these changes are directly related to the injury, yet not ignoring that the presence of psychiatric disorders may interact with cognitive decline (9, 11). In 2017, a study (4) suggested that a specific post-EI syndrome should be recognized and proposed criteria to fit the Diagnostic and Statistical Manual of Mental Disorders (DSM) (12) standards. This proposal points to cognitive decline in auditory and verbal memory dysfunction, visuospatial deficits, word-finding and learning difficulties, and executive function abnormalities.

Changes in cognition are a relevant feature in EI and must be carefully investigated, given that cognitive decline can lead to loss of income, distress, inability to maintain a social life, and trouble maintaining daily activities (4, 8). Neuropsychological changes in EI tend to be delayed (1, 11), worsen over time (7–9), and cause loss of functionality (4, 9), but the understanding of the psychobiological mechanisms involved is still limited (3, 8).

There are still little data about EI, mainly its long-term consequences, although there are some indications about the neuropsychological, psychiatric, and cognitive repercussions of this condition, and there is an ongoing discussion about its inclusion in the DSM. Furthermore, there are no case reports involving the Brazilian population, and there are few reports of cases with an international population, especially among publications in the last 5 years. Considering this, in this case report, we aim to describe a patient with probable neuropsychological deficits of EI and present his profile of cognitive decline and psychiatric symptoms to share the importance of recognizing EI and its profile.

## Methods

The patient was submitted to a complete neuropsychological evaluation to investigate the cognitive decline. The neuropsychological battery included instruments for evaluation of the following domains: memory, attention, language, visuoconstruction, and executive functions. The memory domain was evaluated using the Rey Auditory Verbal Learning Test (RAVLT) (13). Attention was evaluated using the Psychological Battery for Attention Assessment (14). Language abilities were evaluated using the Boston Naming Test (15). The tasks of phonemic verbal fluency (letters F, A, and S) and semantic verbal fluency (category animals) (16) evaluated both language abilities and executive functions. The Dementia Rating Scale-2 (17) was used for evaluating global and specific functioning in all domains evaluated. The clock drawing task was included to evaluate visuoconstruction abilities. The Brazilian version of the Montreal Cognitive Assessment (MoCA-BR) (18) was used as a screening test of general cognitive function, and the Pfeffer Functional Activities Questionnaire (19) was included to evaluate daily living functionality.

Psychiatric symptoms and complaints were evaluated using the Neuropsychiatric Inventory Questionnaire (NPI-Q) (20). In addition, the patient underwent a psychiatric evaluation in conjunction with the neuropsychological evaluation.

All instruments were previously validated for the Brazilian population with a Portuguese version. As to the interpretation of the results, any score below two standard deviations (SD) below the mean (considering patients age, gender, and educational background) or below percentile (Pc%) 5 was considered as a severe impairment in the evaluated domain. In addition, scores below 1 1/2 SD below the mean (considering patients' age, gender, and educational background) were considered an indication of mild impairment. To evaluate the indication of impairment in the clock drawing test, we considered the guidelines proposed by Shulman (21). For the Boston Naming Test, we considered the 12 points cutoff as an indication of impairment. For the MoCA-BR, we considered the cutoff score of 25 points as proposed by Memória et al. (22).

**Figure 1:**
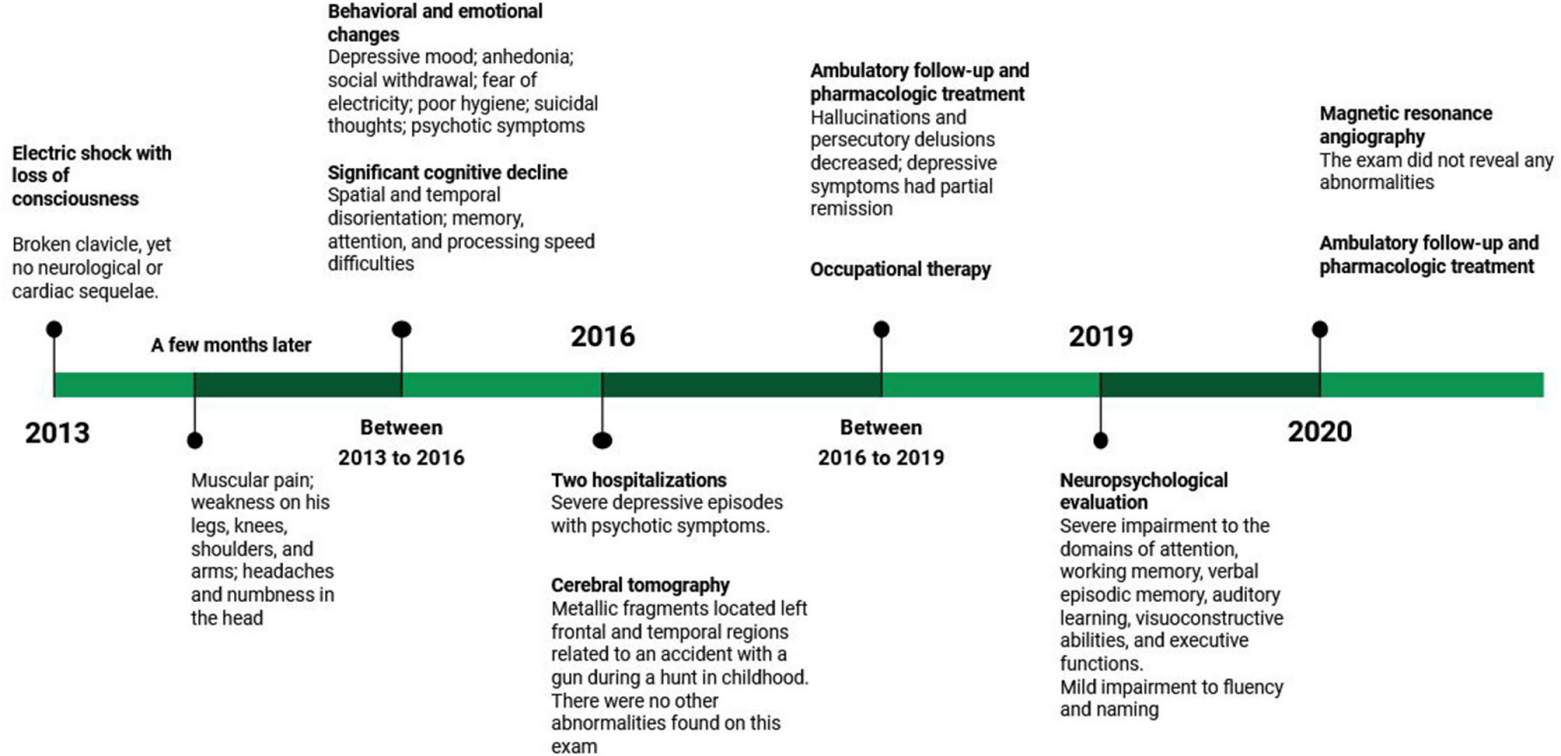
Timeline with relevant data from care.

The patient and his family both provided consent for publication of the following case. Figure 1 presents a timeline with the relevant data from care.

### Patient's Medical History

The patient is a 56-year-old man, right-handed, married, resident in an urban environment, and currently on unpaid medical leave. He had 2 years of formal education and has a low socioeconomic status background. At age 50, he was working as a painter at a pharmacy and touched a bare wire. After the contact with the electrical current, he fell and lost consciousness. He was then immediately taken to a hospital for medical evaluation that revealed a broken clavicle due to the fall, yet no neurological or cardiac sequelae. His wife denied that traumatic brain injury had been diagnosed on this occasion and referred that no other treatment other than the one related to the fracture was performed. A few months after the injury, the patient started to refer to frequent muscular pain and weakness on his legs, knees, shoulders, and arms, and frequent headaches and numbness in the head.

After the accident, the patient presented psychiatric symptoms such as depressive mood, crying episodes, loss of interest in pleasurable activities, social withdrawal, fear of electricity, poor hygiene, and suicidal thoughts. Initially, he reported distressing memories and persistent avoidance of stimuli associated with the traumatic event, which is insufficient for posttraumatic stress disorder diagnosis, and physical exam was normal. During follow-up, as depressive symptoms were present and frequent, causing distress and functional impairment, the diagnosis of major depression was made. These symptoms had partial remission during ambulatory follow-up, which made us change the pharmacological treatment several times. Initially, the patient used sertraline for 4 months [maximum dose (MD), 150 mg], but irritability, anhedonia, social isolation, and sleep disturbance remained. Venlafaxine (MD, 225 mg) replaced sertraline; after a brief period of symptoms intensity reduction and 4 months of use, we changed for amitriptyline (MD, 225mg) because he complained about sadness, anhedonia, loss of energy, social isolation, and irritable mood. For the same reason, we changed the prescription after 11 months of amitriptyline use to fluoxetine (MD, 80 mg). Six months later, bupropion (MD, 300 mg) was added as an adjunctive medication, but the patient achieved again only a partial response (fluoxetine plus bupropion lasts for 4 months). Comparing these medications, venlafaxine gave the best response; another modification in the antidepressants was made (back to venlafaxine in the place of amitriptyline). After 7 months of venlafaxine plus bupropion and olanzapine, the patient stopped all medications. Two months later of bupropion (300 mg), desvenlafaxine (MD, 100 mg), and olanzapine 10 mg, depressive symptoms had the most important reduction; the patient reported occasional sadness and irritable mood.

The patient also presented psychotic symptoms such as auditory hallucinations, which in the beginning were mood-congruent (telling him to commit suicide, for example) and persecutory delusions (somebody is making a plan to damage him). During the follow-up period, the delusion content changed to people inside a black car making a film or spying on him. The patient was hospitalized twice at age 53 for the treatment of severe depressive episodes with psychotic symptoms. Cognitive symptoms were also observed and investigated for possible reversible causes, which were negative. During most of the follow-up, hallucinations and persecutory delusions decreased, which made the patient not act in function of them between April 12, 2018 and October 20, 2020; in this period, olanzapine remained at 10 mg. In the last medical consultation (March 31, 2021), he complained about delusions and how his life was negatively impacted by them, which made us increase olanzapine to 15 mg.

Due to the achievement of only a partial response of depressive symptoms during more than 2 years and periods of psychotic features, it is possible to make the following diagnosis: persistent depressive disorder with persistent major depressive episodes and periods of psychotic features.

During this period, the patient was also followed up fortnightly by occupational therapy to help him perform daily activities and to improve his autonomy, such as assisting with household chores and taking medications alone, for example, besides stimulating cognitive aspects such as attention, mental planning, and memory. However, it showed results below expectations, with significant cognitive and mood fluctuations. In addition to the limitations inherent to dementia, the professional attributes these difficulties to the lack of continuity of the proposed stimulation activities and limited family support.

When hospitalized for treatment of psychiatric symptoms, he underwent a cerebral tomography that revealed metallic fragments located in the left frontal and temporal regions. These fragments are related to an accident with a gun during a hunt in childhood, where he was shot in the head. There were no other abnormalities found on this exam. Unfortunately, when the patient arrived at our service, he no longer had these imaging tests, limiting our analysis to the report described in the medical record. The patient was unable to remember or discuss any details related to the referred accident or treatments performed at the time. His wife was also unable to provide details of the accident other than that it happened when the patient was a child and that it had never interfered with his functionality. In addition, during outpatient clinical follow-up, other clinical examinations were performed to check for reversible causes of dementia, such as syphilis, HIV, hepatitis [anti-hepatitis B (anti-HBs), positive; hepatitis B surface antigen (HBsAg); and anti-hepatitis C virus (anti-HCV), non-reactive], hypothyroidism [thyroid stimulating hormone (TSH), 1.81; T4L, 0.66], blood count [hemoglobin (Hb), 16.8; white blood cell (WBC), 7,130; PLT, 178,000], and renal function [blood urea nitrogen (BUN), 41; Cr, 1.2; Na, 136; K, 5.4; Ca, 10.5], but all were discarded.

**Figure 2:**
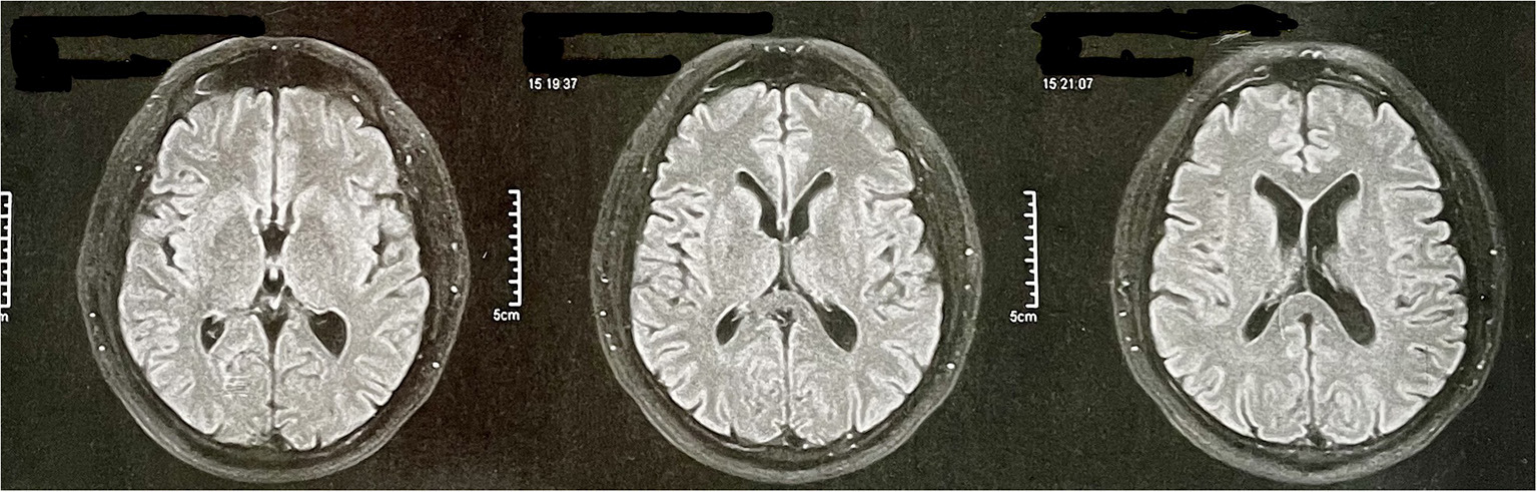
Patient's magnetic resonance angiography data. The magnetic resonance images are restricted to the T2 FLAIR sequence and only axial because they are derived from an angiographic resonance.

At age 56, he underwent a magnetic resonance angiography at the request of his psychiatrist. The magnetic resonance images are restricted to the T2 fluid-attenuated inversion recovery (FLAIR) sequence and only axial because they are derived from an angiographic resonance. The exam did not reveal any abnormalities (Figure 2). At this time, he was still under treatment for behavioral changes and was taking olanzapine and venlafaxine as pharmacological treatment. PET-CT examination was requested, but the patient does not have socioeconomic conditions to perform it.

### Patient's Cognitive Complaints

At the same time that behavioral changes occurred, the patient showed a significant cognitive decline. The patient's family reported spatial and temporal disorientation; difficulty remembering appointments, events, and facts of his life, managing his finances, and participating in family activities; and inability to walk around the neighborhood alone due to the risk of getting lost. He had problems returning to work because he could not pay attention to the commands and took too long to take any action because of difficulties with the cognitive process of processing speed.

He was referred to neuropsychological evaluation at our service at age 55. The patient remained independent in basic activities but depended on his family's help to perform daily activities, such as cooking, managing finances (he did not do previously), performing house chores, and shopping. The patient still presented a depressive mood, social withdrawal, poor hygiene, and loss of interest in pleasurable activities. His wife referred that he presented hyperactive sexual behavior in the form of excessive masturbation. The patient had cognitive difficulties performing daily activities, understanding conversations, and managing several requests at the same time. He had difficulty remembering facts about his life, maintaining coherent speech, and finding words, often taking long pauses or pauses in mid-sentence, referring to not remembering what he was talking about. The patient still reported frequent headaches and muscle weakness.

According to the patient and caregiver's report during the anamnesis interview, before the accident that caused the EI, he did not demonstrate cognitive or behavioral impairments that would interfere with his daily life activities besides not being the manager of the household money due to the low educational level. His wife refers that previous to the injury, the patient was very sociable and hardworking. He was well-known in his community, used to play soccer as a hobby, and was able to help his wife at their small business. After the accident, the patient was unable to maintain his work activities as a painter, becoming a store inspector. However, even so, he was not able to carry out his new professional duties properly, losing his job soon. Over time, he became more and more dependent on family members, demanding continuous care. This led to the overload of the wife, who depends on her work for the financial maintenance of the house and is unable to be present at all times and strictly follow the guidelines of health professionals. In addition, the patient indicated a desire to remain isolated and had been increasingly reducing his social relationships, which is related to dementia and depressive disorder.

## Results

**Figure 3:**
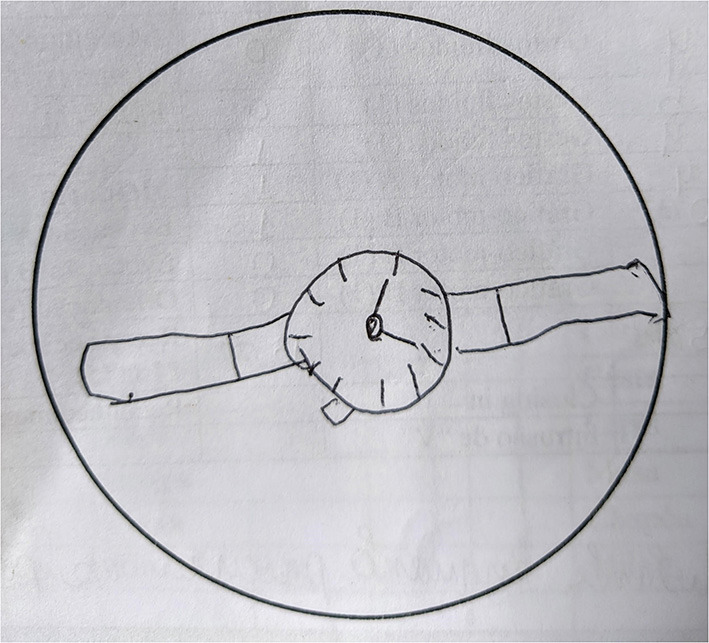
Patient's clock drawing test, showing visuospatial and executive functioning impairment.

The results of the neuropsychological evaluation revealed severe impairment to the domains of attention, verbal episodic memory, working memory, auditory learning abilities, visuoconstructive abilities (Figure 3), and executive functions, and a mild impairment to fluency and naming (see Table 1). Based on the clinical symptoms described above, we presented the neuropsychological and psychiatric consequences of EI. Now, we are evaluating the prescription of a cholinesterase inhibitor because cognitive complaints persist during all follow-up, which still harms the patient's life.

**Table 1:** Results of the neuropsychological battery.

**Instruments applied**	**Result**	Score/percentile^*^
Montreal cognitive assessment	6/30	Cutoff 25
Boston naming test	11/15	Cutoff 12
Semantic fluency test	4 words	−1.72
Phonemic fluency test	4 words	−1.66
Clock drawing test	2/5	
Rey auditory verbal learning test		
• ΣA1A5	17/75	Pc% <5
• A7	0–15	Pc% <5
• Recognition	−6/15	Pc% <5
Psychological battery for attention assessment—total score	−14/360	Pc% 1
• Focused attention test	1/120	Pc% 1
• Divided attention test	−5/120	Pc% 1
• Alternating attention test	−10/120	Pc% 1
Dementia rating scale-2—total score	87/144	−10.77
• Attention	30/37	−5.7
• Initiation/Perseveration	13/37	−31.40
• Construction	3/6	−3.50
• Conceptualization	30/39	−1.72
• Memory	11/25	−5.58
Neuropsychiatric inventory questionnaire—total score	15/36	
• Delusions	2/3	
• Hallucinations	0/3	
• Agitation/Aggression	0/3	
• Dysphoria/Depression	2/3	
• Anxiety	3/3	
• Euphoria/Elation	0/3	
• Apathy/Indifference	2/3	
• Disinhibition	0/3	
• Irritability/Lability	2/3	
• Aberrant motor	2/3	
• Nighttime disturbances	2/3	
• Appetite/Eating	0/3	
• Disturbances	0/3	
Pfeffer functional activities questionnaire	18/30	

* *Scores of all cognitive tests indicated neurocognitive impairment according to published normative data for standardized tests*.

## Discussion

The patient fulfilled all criteria for dementia according to the fifth edition of the Diagnostic and Statistical Manual of Mental Disorders (DSM-5) (12). Our evaluation revealed evidence of cognitive decline severe enough to interfere with his independence in daily activities. Besides the impaired performance at the neuropsychological evaluation, the patient's wife provided reliable evidence to corroborate the significant decline of the patient's previous levels of functioning. The consideration of both the neuropsychological results and the patient's history led to the conclusion that this was a major neurocognitive disorder.

Initially, the behavioral variant of frontotemporal dementia (bvFTD) was considered, as it is a common cause of young-onset dementia. In addition, it has relevant characteristics related to behavioral and personality changes (23, 24). Yet, in addition to not meeting the minimum criteria established by the International Behavioral Variant FTD Criteria Consortium (25), the patient had severe impairments in cognitive domains—such as memory and spatial disorientation—from the beginning, which are considered exclusion criteria for bvFTD (24). The speech alterations presented by the patient during the evaluation also stood out, leading to an investigation of the linguistic variants of FTD. As the neuropsychological results did not identify significant losses in the domain of language, and as the cognitive and behavioral changes were very prominent from the beginning—and being this is a less intense characteristic in the linguistic variants of FTD—we discard this possibility. Finally, FTD was discarded because the image examination does not indicate any type of cortical atrophy (Figure 2).

Due to the indication of the psychiatrist who referred the patient for neuropsychological evaluation, we also examined the chance of pseudo-dementia, which was soon ruled out. Although there are no well-established criteria for this condition, it is known that pseudo-dementia is a psychiatric condition that disguises itself as a neurodegenerative disease, being reversible when the psychiatric condition is successfully resolved or treated (26). In this case, the cognitive symptoms did not decrease after pharmacological intervention, and other conditions can better explain symptoms. The possibility of reversible causes of dementia, such as syphilis, HIV, and hypothyroidism, and dementias influenced by bodily functions, such as kidney and liver function, was also investigated, but none of the clinical tests indicate significant changes. There is also no indication that it is dementia of degenerative etiology since the results of the cognitive screenings [Mini-Mental State Examination (MMSE)], carried out by the psychiatric team that has been following the patient since 2017, indicate a stabilization of cognitive impairments (see Supplementary Table 1). Besides, the qualitative reports of the occupational therapist who has been accompanying him fortnightly since 2018 also do not demonstrate the existence of cognitive worsening, but eventual fluctuation.

Given the patient's history of an electrical shock before the cognitive complaints, we researched the topic. This research led us to find studies that describe deficits in episodic memory, attention, working memory, word-finding abilities, executive function, visuospatial, and motor abilities (4, 8–10) as common in EI cases. We further considered the proposal of Andrews et al. (4) of postelectric or lightning injury syndrome (PELIS) as criteria for differential diagnosis. Physical findings are present in the case, such as frequent muscular weakness, numbness, and pain.

Our patient presents key neuropsychological elements, with severe executive and memory impairment. Executive elements include impairment in processing speed, executive ability, attention, and auditory learning. The patient also presents impairment in episodic memory, working memory, and word-finding difficulties.

Depressive symptoms can be a part of PELIS or can be a part of an associated psychiatric disorder. Our patient had a consistent depressive mood, loss of energy, apathy, loss of interest in pleasurable activities, and social withdrawal that decreased but did not cease with pharmacological intervention. He had no history of depression and did not report having any of these symptoms previously to the electrical shock. The patient's wife said he had a consistent fear of electricity since the electrical shock episode, yet there was no phobia diagnosis. These features are a part of the four miscellaneous symptoms frequently present in PELIS cases (4). Neuropsychological evaluation was important for the differential diagnosis of this case. The alterations observed in the neuropsychological battery and the persistence of patient cognitive complaints contributed to the cholinesterase inhibitor prescription idea. There is no literature evaluating this use, but for cognitive decline due to traumatic brain injury, some papers report the prescription of these medications to reduce commitment in memory, attention, and executive function (27, 28).

Executive dysfunction is the most striking feature of this case. The decline in working memory, processing speed, inhibitory control, and attention strongly decreased the patient's functional abilities and caused great interpersonal and financial distress. Due to the inability of following plans and attending requests in the workspace, the patient was required to take a medical leave. The psychiatric alterations presented also contributed to the reduction in the patient's functionality. The sum of all these findings leads us to conclude that this was, in fact, a case of EI with strong neuropsychological symptoms, fitting criteria for PELIS as proposed by Andrews et al. (4).

Considering these features, the patient was referred to neuropsychological rehabilitation focusing on improving his functionality and regaining independence. He was also referred to neurological evaluation for further investigation of the symptoms of numbness and muscular pain. He also continued psychiatric treatment and occupational therapy.

We understand that one main limitation of this study was that it was not possible to carry out more in-depth investigations using functional neuroimaging and/or genetic tests due to the financial difficulties of the patient and our service, and the impossibility to access details related to the gun accident that led to the metallic fragments in the left frontal and temporal regions, the following treatments performed at the time and cerebral tomography results. These facts add to the complexity of the case difficulty in the elimination of other possible diagnostic hypotheses, requiring a much more thorough clinical analysis. Yet, we believe that this case report is relevant, as it shares the importance of recognizing EI and its profile, especially in the Brazilian context, where we do not find studies of this type. We understand that the proper diagnosis of cases like this can lead to better management and interventions of the disease, reducing the impacts on the quality of life of the patient and his family. We argue that this condition should be considered by clinicians whenever a patient survives an accident with electric shock or lightning. However, the poor recognition of this injury may be due to the absence of official criteria for this condition in diagnostic manuals, such as the DSM-5 (4). Even though this is a very unique case, with a specific patient profile that cannot be generalized, we understand that sharing these findings can help other professionals in dealing with similar cases.

## Data Availability Statement

The original contributions presented in the study are included in the article/Supplementary Material, further inquiries can be directed to the corresponding author/s.

## Ethics Statement

Written informed consent was obtained from the individual(s) for the publication of any potentially identifiable images or data included in this article.

## Author Contributions

KMA was involved in the design of the study, supervised and reviewed the neuropsychological assessment, and critically reviewed the manuscript. JPA performed the neuropsychological assessment, was involved in the study design and writing of the paper. MS contributed to the writing of the article. WS contributed to the psychiatric evaluation and analysis of functional neuroimaging, and critically reviewed the manuscript. All authors contributed to the article and approved the submitted version.

## Conflict of Interest

The authors declare that the research was conducted in the absence of any commercial or financial relationships that could be construed as a potential conflict of interest.

## Abbreviations

bvFTD: behavioral variant of frontotemporal dementia
DSM: Diagnostic and Statistical Manual of Mental Disorders
EI: electrical injury
FTD: frontotemporal dementia
PELIS: postelectric or lightning injury syndrome.
