# Supplementary material for: Case Report in the Brazilian Context: Cognitive and Behavioral Changes Following an Electric Injury
Source: Front Psychiatry. 2021 Jul 20;12:684817. doi: 10.3389/fpsyt.2021.684817 (PMC8331101; doi:10.3389/fpsyt.2021.684817)
Supplement: Supplementary file 1 [file Table_1.docx]

**Supplementary Table 1:** Results of the MMSE during psychiatric follow-up

| Date | Instruments applied | Score |
| --- | --- | --- |
| 08/09/2018 | Mini-Mental | 11/25 |
| 01/09/2019 | Mini-Mental | 14/25 |
|  | - Temporal orientation | 02 |
|  | - Spatial orientation | 04 |
|  | - Registration | 02 |
|  | - Attention and calculation | 00 |
|  | - Remote memory | 00 |
|  | - Naming | 02 |
|  | - Speech | 01 |
|  | - Stage command | 03 |
|  | - Reading and obey | 00 |
|  | - Writing a complete sentence | 00 |
|  | - Copying | 00 |
| 01/09/2019 | Fluency (animals) | 07 |
| 01/09/2019 | Clock Drawing Test | 00 |
| 02/05/2020 | Mini-Mental | 13/25 |
|  | - Temporal orientation | 02 |
|  | - Spatial orientation | 03 |
|  | - Registration | 02 |
|  | - Attention and calculation | 00 |
|  | - Remote memory | 00 |
|  | - Naming | 02 |
|  | - Speech | 01 |
|  | - Stage command | 03 |
|  | - Reading and obey | 00 |
|  | - Writing a complete sentence | 00 |
|  | - Copying | 00 |
| 02/05/2020 | Clock Drawing Test | 00 |
| 02/05/2020 | Basic ADLs | 06/06 |
| 02/05/2020 | Instrumental ADLs | 03/08 |
